# Accuracy of lingual straight-wire orthodontic treatment with passive self-ligating brackets and square slot: a retrospective study

**DOI:** 10.1186/s40510-023-00482-3

**Published:** 2023-09-18

**Authors:** Fabrizio Scisciola, Mario Palone, Giuseppe Scuzzo, Giacomo Scuzzo, Luis T. Huanca Ghislanzoni, Luca Lombardo

**Affiliations:** 1 Postgraduate School of Orthodontics, University of Ferrara, Ferrara, Italy; 2 Department of Orthodontics, Dental School, University of Geneva, 1205 Geneve, Switzerland

**Keywords:** Lingual straight-wire, Digital set-up, Self-ligating lingual appliances, Square slot

## Abstract

**Objectives:**

To investigate the accuracy of torque, tip and rotation and linear intra-arch movements yielded by passive self-ligating lingual straight-wire appliances with brackets featuring square slots.

**Materials and methods:**

Twenty-five adult Caucasian patients (16 females and 9 males; mean age 26.5 ± 4.3 years) with Class I or mild Class II head-to-head malocclusion were orthodontically treated via passive lingual self-ligating straight-wire appliances (ALIAS, Ormco, Orange, CA) with no extraction. Records were retrospectively analysed, and digital models of pre-treatment (*T*0), planned (*T*1) and achieved (*T*2) phase were acquired for both arches in each patient via an intraoral scanner (Medit I500 (iScan Medit, Seoul, Korea). VAM software (Vectra, Canfield Scientific, Fairfield, NJ, USA) was used to measure both angular values (torque, tip and rotation) and linear intra-arch widths (between canines, first and second premolars and first and second molars). Measurements were obtained for all the movements investigated for each tooth group (incisors, canines, premolars and molars), by arch (maxillary and mandibular) and for both arches at *T*0, *T*1 and *T*2. The accuracy of angular values was compared using Student's *t*-test against a hypothetical 100%, and among the various tooth groups by post-hoc tests. Transverse linear measurements were investigated by means of the non-parametric Friedman test. The significance threshold was set at 0.05.

**Results:**

The mean accuracy of angular values was 77.25 ± 7.71% for torque, 78.41 ± 6.17% for tip and 77.99 ± 6.58% for rotation. In all cases, however, there was a significant difference between planned and achieved movements, and accuracy was significantly lower than the hypothetical 100% for all tooth groups, individual arches and dentition (*p* < 0.001). For intra-arch diameters, the greatest accuracy values were found for the anterior sectors (83.54 ± 5.19% for the maxillary inter-canine distance) and the lowest for the posterior sectors (67.28% for the maxillary inter-second molar distance).

**Conclusion:**

Straight-wire lingual treatment with passive self-ligating appliances featuring with square slot displayed excellent clinical accuracy, albeit with statistical accuracy decreasing antero-posteriorly.

## Background

Nowadays clear aligners are the most common orthodontic device, due to their aesthetic properties [1] and their high acceptance by patients, both adults and adolescents [2]. Despite their considerable diffusion, their clinical indication is for the treatment of non-extractive orthodontic cases of mild to moderate difficulty [3]; orthodontic cases requiring major root torque movements [4], bodily translation in extraction cases [5], severe rotations of rounded teeth [6] and over-bite corrections ≥ 1.5 mm [7], on the other hand, should be addressed by means of fixed appliances [8].

In complex cases where an aesthetic treatment is requested, the fixed lingual appliances can be considered the orthodontic device of choice, especially for adult patients [9]. In fact, from a biomechanical perspective, albeit with some differences [10], the lingual appliance is clinically comparable to the vestibular one [11, 12].

Beginning in the 1980s–90 s, the lingual technique has undergone significant improvements due to our better understanding of appliance biomechanics and to significant improvements in metallurgy, which have allowed on the one hand the miniaturization of lingual brackets [13] and on the other the introduction of orthodontic archwires made of copper–nickel–titanium alloys (Cu–NiTi), with shape memory and superelastic properties [14]. Indeed, the miniaturization of lingual brackets has enabled the inter-bracket distance to be increased, accompanied by a consequent increase in the elasticity of the orthodontic archwire [15]. It also means that the bottom of the slot can be positioned as close as possible to the lingual surface, enabling tooth height errors attributable to torque to be minimized [16]. A further advantage of smaller brackets is that the appliance is more comfortable for the patient [13].

In 2011, the culmination of the evolution in orthodontic brackets led to the introduction of the first passive lingual bracket with 0.018 × 0.018-in. square slot, i.e., with the presence of four rigid walls [17]. Assuming the precise replication of the edges of the wire and the slot, following the use of a full-thickness orthodontic archwire, the wire-slot play for both second and third order information is reported to be equal. In addition, the square slot is more efficient than the traditional rectangular slot in correcting rotations, with both round and square archwires, since the first order wire/slot play is significantly decreased [17].

A further aspect to underline is that the presence of the fourth wall ensures that the archwire remains engaged within the slot during the correction of tooth derotations or the retraction of the anterior sector in extraction cases [17], with consequent minimum loss of torque (vertical bowing effect) and therefore a better three-dimensional control of the teeth [18]. Moreover, the ligation method is not operator-dependent, but presupposes a progressive filling, both horizontally and vertically, of the slot, up to the use of a full-thickness orthodontic archwire [13].

That being said, the remarkable manufacturing precision of the slot and archwire requires the execution of a precise set-up, which is always necessary in lingual technique for a number of reasons. First of all, the considerable lack of homogeneity of the lingual surfaces always makes it necessary to customize the lingual appliance. In the case of the lingual straight-wire technique, customization is performed at the level of the orthodontic bracket bases with the creation of differential composite thicknesses [19]. Furthermore, the customization of lingual appliances must allow for any dental overcorrection, typically necessary in extraction cases [20].

All this is possible thanks to the technological advances that began in the early 2000s and now allow the execution of a digital set-up [21]. Modern digital technologies have not only made this procedure more efficient and less laborious than manual set-up, but also enable precise measurement of a series of variables, including the height of the brackets, which is easily replicable on the right and left sides. In addition, any changes and overcorrections can be performed instantly and with considerable ease, as compared to manual set-up. Moreover, storage needs are eliminated and remote communication between clinicians is facilitated [22].

Although other authors have investigated the clinical efficacy of lingual appliances [23–26], no one has yet investigated the clinical efficacy of orthodontic was therefore to investigate the clinical accuracy of such appliances with respect to the results planned in the digital set-up.

## Materials and methods

This retrospective study was approved by the University of Ferrara Ethics Committee, and the protocol registered as number 7/2022. The sample size was calculated in a study to validate the measurement method used [27], in a similar fashion to that reported by Albertini et al., who determined a minimum sample of 24 patients [23]. Therefore, 25 adult patients of Caucasian origin (16 females and 9 males; mean age 26.5 ± 4.3 years) who had undergone non-extractive orthodontic treatment with ALIAS passive self-ligating lingual straight-wire appliances (Ormco, Orange, Cal, USA) were selected retrospectively. All patients had been treated at a private clinical practice by a single operator (GS), expert in the lingual technique.

The retrospective selection of patients involved the following inclusion criteria:

- Adults with complete permanent dentition
- Subjects suffering from Class I malocclusion or mild Class II head-to-head malocclusion whose treatment involved the use of Class II elastics for no more than 4 months
- Presence of slight crowding in both arches (≤ 3 mm)
- Subjects undergoing non-extractive orthodontic treatment
- Absence of shape anomalies, supernumerary teeth, systemic pathologies and pharmacological treatments that hinder or may influence orthodontic movement

### Appliance customization and clinical procedures

For each patient, the following diagnostic records were acquired: intraoral photos, extraoral photos, x-rays (panoramic radiograph and cephalogram) and pre-treatment digital models (*T*0), constructed using the Medit I500 intraoral scanner (iScan Medit, Seoul, Korea). The customization of the lingual equipment took place after the execution of the digital set-up using the proprietary software (ELINE system software, Dijiset Sas-Digital Medical Company, Rome, Italy). The aims of the treatment were to obtain aligned arches with canine and molar Class I ratios, centred midlines and adequate overjet and overbite (1–3.5 mm). No overcorrections were included in the set-up.

**Fig. 1:**
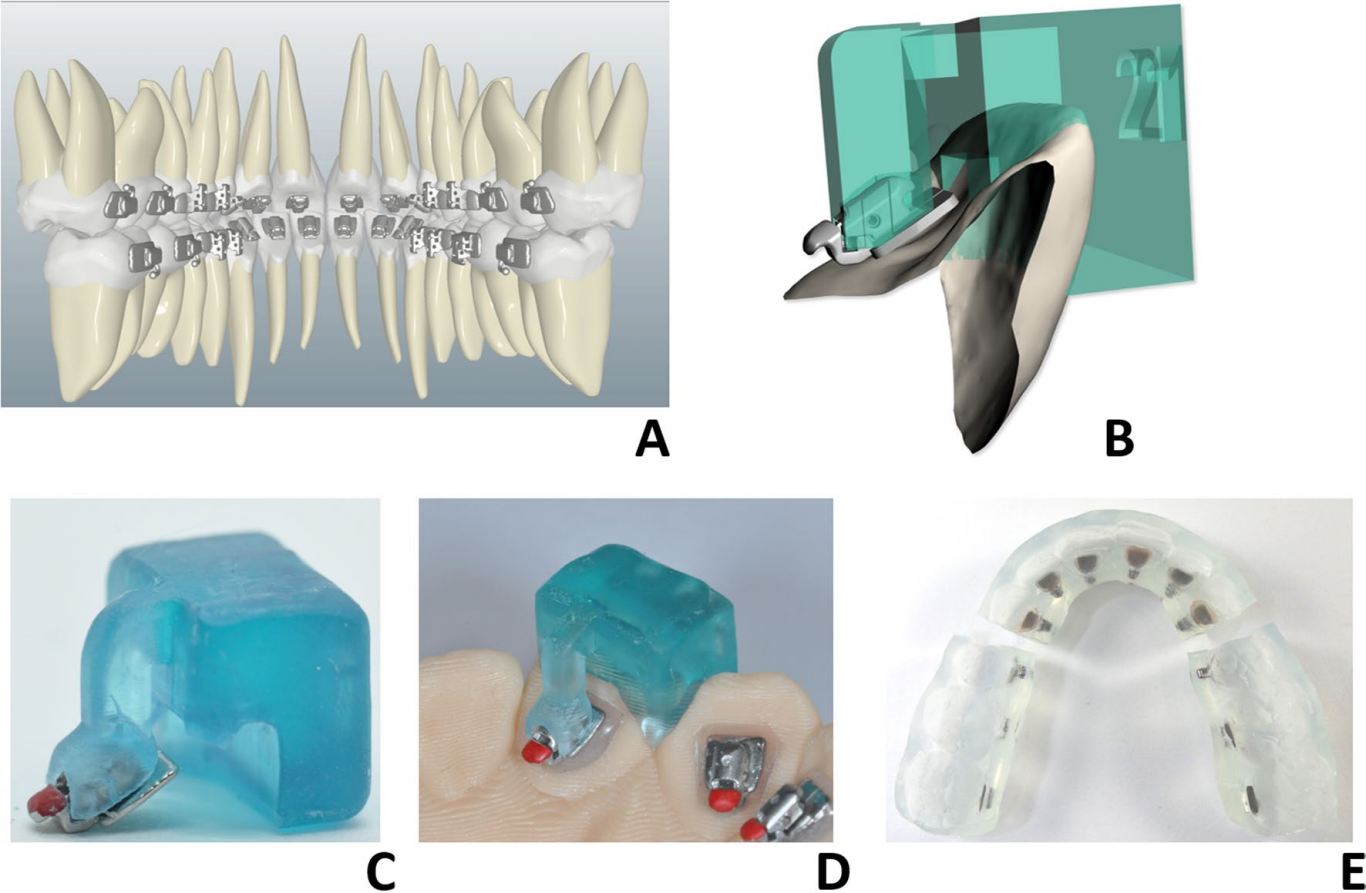
Positioning of the lingual brackets with respect to the lingual straight plane (LSP) (**A**), digital design of the transfer jig (**B**), positioning of the lingual bracket in the prototyped jig (**C**) with positioning of the latter on the malocclusion model (**D**). Finally, creation of the transfer tray in transparent silicone (**E**)

Bracket positions were planned digitally, adhering to the positioning with respect to the lingual straight-wire plane (LSP) identified for each arch [28]. In particular, the centre of the self-ligating bracket slot was to sit at the level of half the lingual coronal height in the posterior and anterior sectors (canine to canine) in the mandible, at the level of a third the gingival height of the lingual clinical crown in the anterior maxilla (canine to canine) and at half the palatal coronal height in the posterior maxilla (Fig. 1A).

The software uses a specific algorithm to design the transfer jigs for each individual tooth (Fig. 1B); these were printed using a DPL technology 3D printer (Nexdent 5100, 3D System, Rock Hill, USA) at high resolution (*Z* axis = 50μ). Each lingual bracket was inserted into the respective jig (Fig. 1C), and then the latter was positioned on the malocclusion model (Fig. 1D). In this phase, any gap between the bracket base and the lingual surface of the corresponding tooth was filled with flowable composite (LV Pink Kommonbase, GC Orthodontics Europe GmbH, Breckerfeld, Germany). Once all the brackets and tubes had been positioned on the malocclusion model, a transparent silicone transfer tray (Finopaste Crystal, Fino GmbH, Germany) was created for the purposes of indirect bonding (Fig. 1E). Clinical lingual bonding was performed by a single operator (GS) using light-cured flowable composite (HV Clear Kommonbase, GC Orthodontics Europe GmbH, Breckerfeld, Germany).

**Fig. 2:**
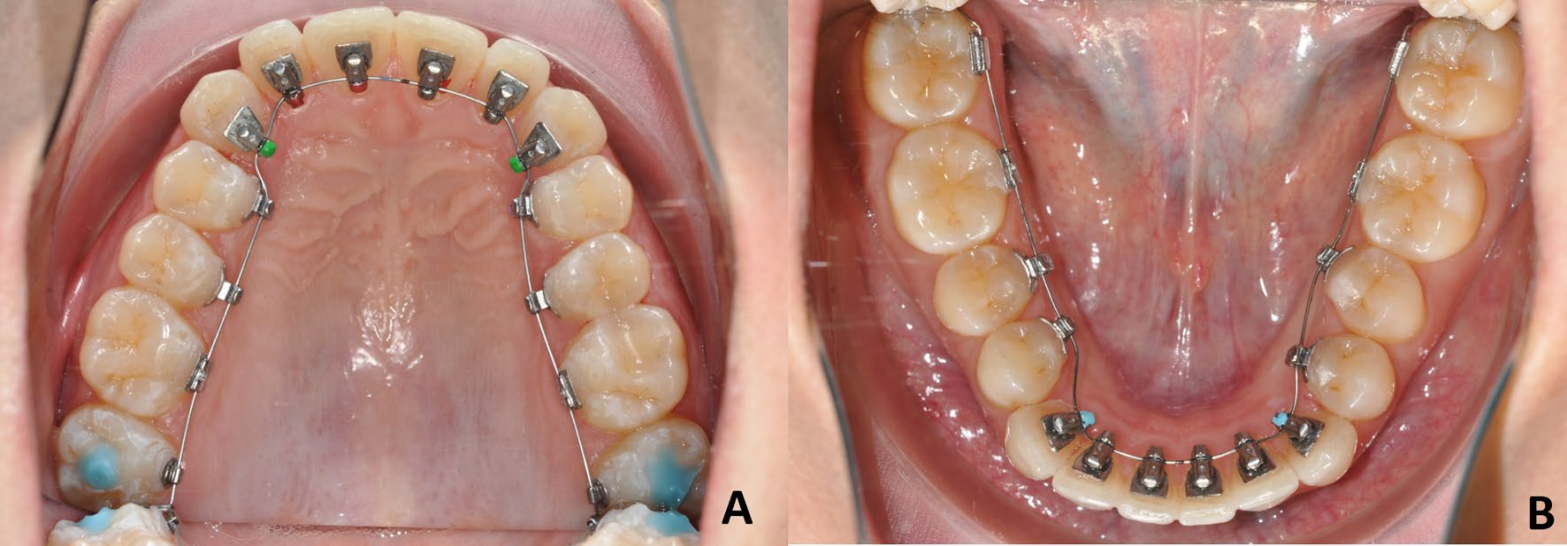
End of the clinical phase of indirect bonding in both the maxillary (**A**) and mandibular (**B**) arch

The same operator (GS) treated each patient using the same archwire sequence on both arches, namely: 0.013-in. and 0.016-in. Cu–NiTi in the alignment phase, followed by 0.016 × 0.016-in. and 0.018 × 0.018-in. Cu–NiTi in the levelling phase and, finally, 0.0175 × 0.0175 titanium-molybdenum alloy (TMA) wire in the detailing phase (Fig. 2A, B). Clinical procedures such as interproximal enamel reduction or IPR (≤ 3 mm) and the use of Class II elastic bands (6.5 oz; 5/16-in.) were allowed for a period not exceeding 4 months.

The mean duration of orthodontic treatment was 18.3 ± 4.3 months. At the end of the treatment, digital post-treatment models (*T*2) were acquired using the same Medit I500 intraoral scanner (iScan Medit, Seoul, Korea), and the digital set-up models (*T*1) were extrapolated directly in STL format using the proprietary ELINE software.

### Measurement of digital models

**Fig. 3:**
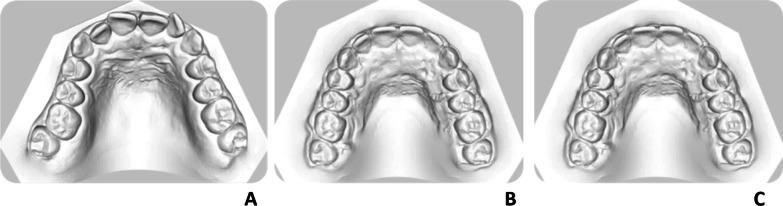
Digital models investigated: pre-treatment (**A**), set-up (**B**) and post-treatment (**C**)

Digital models pertaining to each single subject in each group were analysed by a single operator (FS) using VAM® software (Vectra, Canfield Scientific Inc., Fairfield-New Jersey, USA), adopting the method proposed by Huanca Ghislanzoni (Huanca Ghislanzoni LT. 2013(27)). Measurements were made on pre-treatment (*T*0), set-up (*T*1) and post-treatment digital models (*T*2) (Fig. 3A–C). In brief, 100 anatomical reference points per model were identified, including second molars, and their three-dimensional coordinates exported into specific.txt files (Microsoft Excel®, Microsoft, Redmond, WA, USA). This enabled extrapolation through a complex algorithm of the tip, torque and in–out values of each tooth with respect to an occlusal reference plane passing through the following points:

- The mesiovestibular cusp on the right first molar (Point A)
- The mesiovestibular cusp on the left first molar (Point B)
- The centroid of all the most occlusal points on the FACC line (the facial axial of the clinical crown) of all teeth, excluding the cusp of the canines and the second molar.

**Fig. 4:**
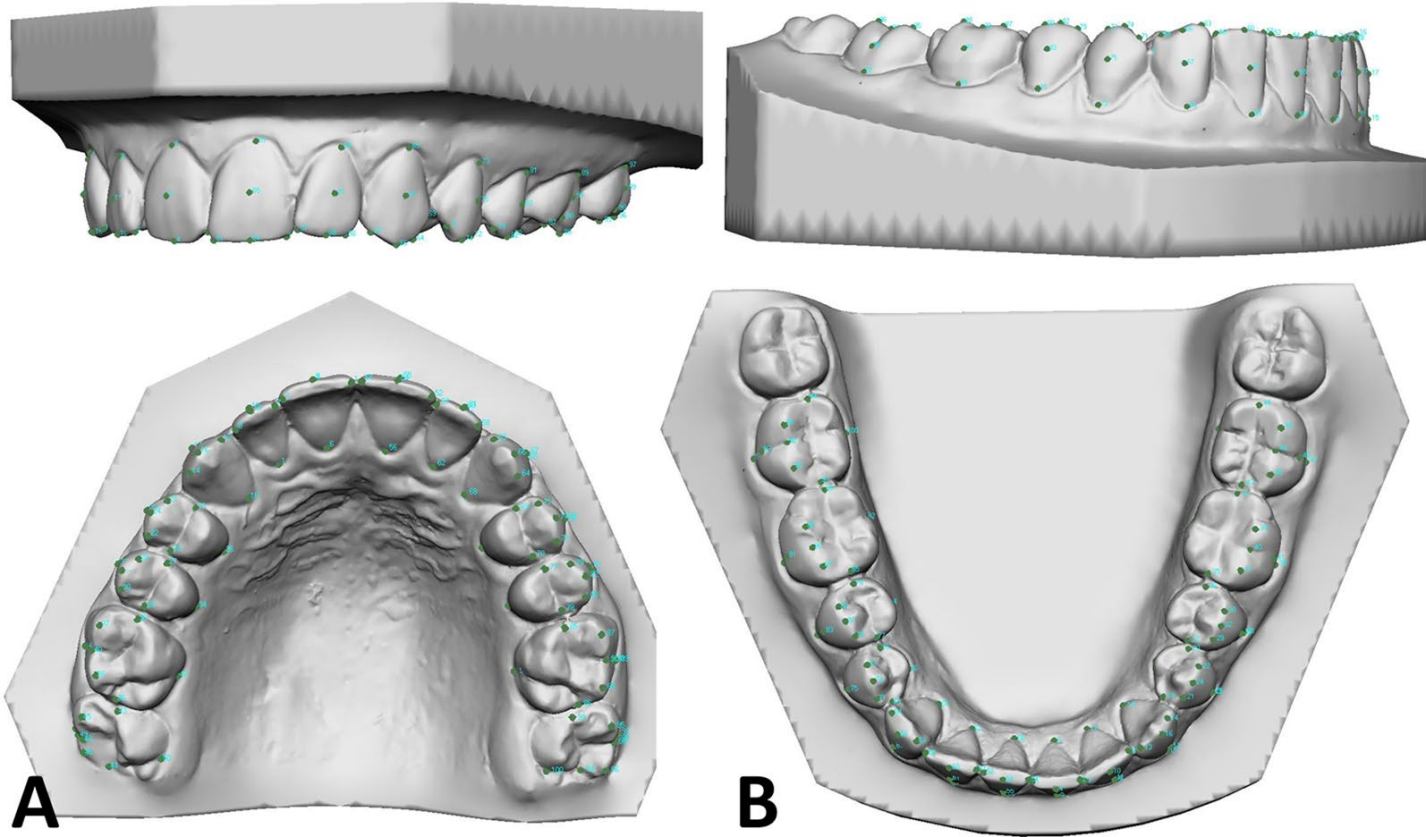
Positioning of 100 anatomical points in both maxilla (**A**) and mandible (**B**)

Thus, six points were assigned to the incisors and canines, respectively, and eight points were assigned to each of the premolars and molars (Fig. 4A, B).

### Tip, torque and in–out measurement

**Fig. 5:**
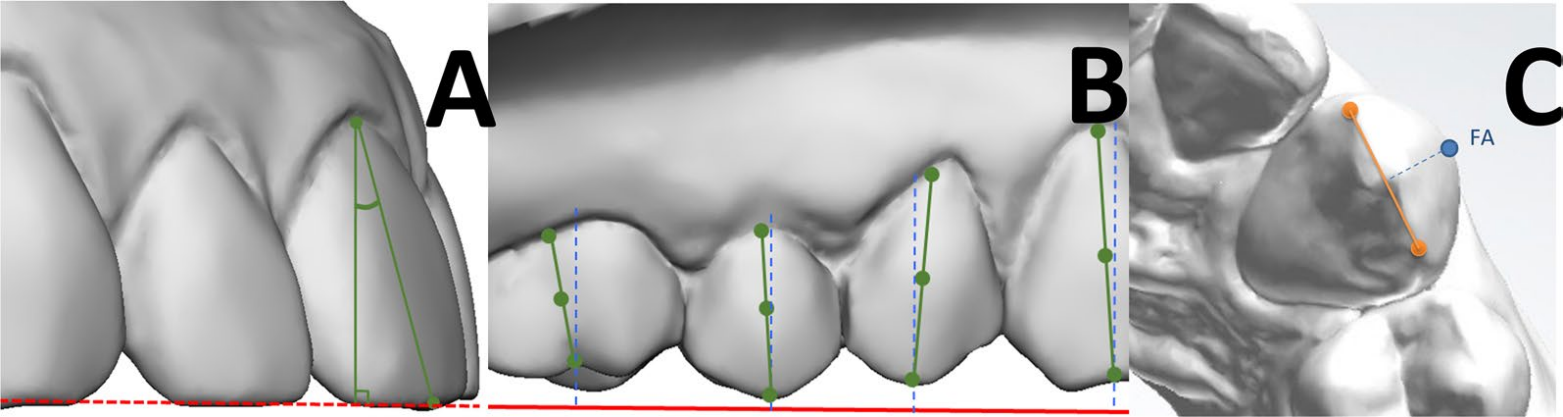
Graphical representation of torque (**A**), tip (**B**) and rotation (**C**) measurements

Torque was measured as the labiolingual inclination (Fig. 5A), and tip as the mesiodistal inclination of the FACCs relative to the occlusal reference plane (Fig. 5B). An individual tooth coordinate system was necessary to determine such values. In–out was measured considering the distance between the FA point and the mesial and distal points of the buccal ridge of each tooth (Fig. 5C).

### Linear measurements

**Fig. 6:**
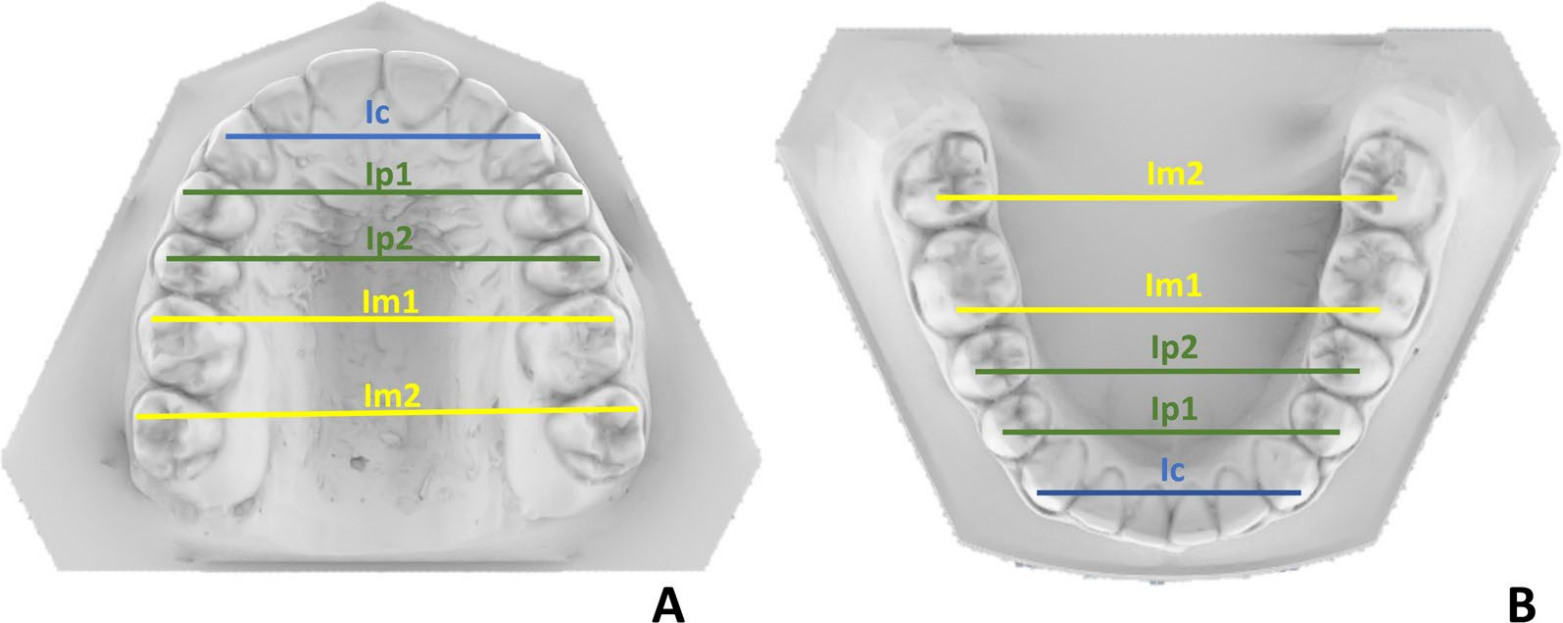
Graphical representation of transverse linear intra-arch measurements in maxilla (**A**) and mandible (**B**). IC: inter-canine width; IP1: inter-first premolar width; IP2: inter-second premolar width; IM1: inter-first molar width; IM2: inter-second molar width

The transverse linear measures calculated for each arch were as follows:

- Inter-canine width (IC): linear distance measured between the tip of the cusps of the canines
- Inter-premolar 1 width (IP1): linear distance between the top of the vestibular cusps of the first premolars
- Inter-premolar 2 width (IP2): linear distance between the top of the vestibular cusps of the second premolars
- Inter-molar 1 width (IM1): linear distance between the top of the vestibular cusps of the first molars
- Inter-molar 2 width (IM2): linear distance between the top of the vestibular cusps of the second molars (Fig. 6A, B)

### Reliability of measurements

To test intra-operator repeatability, 25% of all digital models (12 patients) were randomly selected, and measurements were repeated by the same operator after four weeks. The method error (ME) was calculated according to Dahlberg’ formula, and Wilcoxon *t*-test was used to assess any systematic error (SE) between the two sets of measurements (considering both linear and angular measurements), with significance threshold set at *p* value < 0.05.

The main systematic error value was 0.616, with no value < 0.05 detected; the main method error was 0.117° for angular values and 0.053 mm for linear values, and statistical analysis confirmed the reliability and repeatability of the measurements performed.

### Statistical analysis

Descriptive statistical analyses (*n*. observations, mean and standard deviation (SD)) were performed for the three time-points examined (*T*0, *T*1 and *T*2); for angular measurements (torque, tip and rotation), each tooth group in both arches (incisor, canine, premolar and molar), the single arches (maxilla and mandible) and for both. For linear measurements, (IC, IP1, IP2, IM1 and IM2), the two arches (maxilla and mandible) were considered separately. For both measurements, the imprecision, i.e., the difference between *T*2 and *T*1, was also calculated (|*T*2–*T*1|).

In addition, the accuracy of each movement investigated was calculated, i.e., the percentage of linear or angular movement achieved (real) with respect to that planned (ideal) according to the formula:

$$\begin{aligned} {\text{Accuracy }} &= \, \left[ {{\text{Achieved }}\left( {T{2}{-}T0} \right) \, /{\text{ Planned }}\left( {T{1}{-}T0} \right)} \right] \\& \quad \times 100 \end{aligned}$$

If the movement achieved (*T*2–*T*0) were equal to that planned (*T*1–*T*0), their ratio would be equal to 1, indicating 100% clinical accuracy.

For angular movements, accuracy was compared to a hypothetical 100% using the single-sample Student *t*-test, as was the comparison between achieved and planned movements. Any differences in accuracy among the individual tooth groups was subsequently investigated. First, the Levene test was used to investigate the homogeneity of variance; if this was not significant, the ANOVA test would be applied, or otherwise, the robust version of Brown–Forsythe’s ANOVA would be used to test the null hypothesis of equality between the averages. In the event of one of the two tests yielding a significant result, indicating that there was at least one significant difference between the various pairs, the individual groups would be subjected to pairwise comparison by Fisher’s least-significant difference (LSD) post-hoc test or Tamhane’s post-hoc test, respectively.

For linear measurements, the non-parametric Friedman test was performed to verify whether there were statistically significant differences in the five linear measurements examined (IC, IP1, IP2, IM1 and IM2) at time-points *T*0, *T*1 and *T*2 for both the maxillary and mandibular arches. If the result was statistically significant, pairwise comparisons were made to identify any differences between *T*0, *T*1 and *T*2.

A significance threshold of 0.05 was used for all statistical analyses.

## Results

### Torque

In all cases, there was a significant difference between planned and achieved torque (*p* < 0.001). The average total accuracy was 77.25% ± 7.71%, while the accuracy values for each individual tooth group ranged between a maximum of 82.98% ± 4.64% (maxillary incisors) and a minimum of 69.84% ± 7.29% (mandibular molars). Comparison of the accuracy of the torque movement achieved with a hypothetical 100% was always statistically significant (*p* < 0.001) (Table 1).

**Table 1:** Mean and SD of angular torque values for the planned (*T*1–*T*0), the achieved (*T*2–*T*0), the imprecision (|*T*2–*T*1|) and the accuracy (%) considering the individual dental groups, the individual jaws (maxilla and mandible) and the total

Arch	Tooth group	N. observations	Torque									
			Planned (*T*1–*T*0)		Achieved (*T*2–*T*0)		Planned versus achieved	Imprecision l*T*2–*T*1l		Accuracy		Versus 100%
			Mean (°)	SD (°)	Mean (°)	SD (°)	*p* value	Mean (°)	SD (°)	Mean (%)	SD (%)	*p*-value
Maxilla	Incisor	91	10.25	16.86	8.67	14.34	< 0.001*	2.43	1.91	82.98	4.64	< 0.001*
	Canine	45	4.19	18.13	3.67	15.56	< 0.001*	2.09	1.82	82.89	5.41	< 0.001*
	Premolar	88	1.72	18.18	1.54	14.93	< 0.001*	2.52	2.41	77.97	5.49	< 0.001*
	Molar	90	− 8.32	20.53	− 5.33	15.61	< 0.001*	4.63	3.76	68.72	7.06	< 0.001*
Mandible	Incisor	73	5.56	10.54	4.49	8.67	< 0.001*	1.91	1.07	81.24	3.81	< 0.001*
	Canine	44	6.83	6.73	5.63	5.64	< 0.001*	1.47	0.78	81.43	4.07	< 0.001*
	Premolar	85	1.33	9.27	1.08	7.24	< 0.001*	1.78	1.05	77.61	4.64	< 0.001*
	Molar	78	4.56	6.83	3.08	4.65	< 0.001*	2.25	1.51	69.84	7.29	< 0.001*
Maxilla		314	1.66	19.76	1.94	15.92	< 0.001*	3.04	2.86	77.48	8.24	< 0.001*
Mandible		280	4.21	8.86	3.24	6.99	< 0.001*	1.91	1.19	76.99	7.05	< 0.001*
Total		594	2.86	15.64	2.55	12.54	< 0.001*	2.51	2.31	77.25	7.71	< 0.001*

*The mean value of accuracy was compared with a hypothetical 100% (*p* < 0.05 considered as significant)

### Tip

Similarly, in all cases, there was a significant difference between planned and achieved tip (*p* < 0.001). The average total accuracy was 78.41% ± 6.17%, while for each individual tooth group it ranged between a maximum of 80.72% ± 6.34% (maxillary incisors) and a minimum of 77.42% ± 7.29% (mandibular canines). Comparison of the tip accuracy with respect to a hypothetical 100% was always statistically significant different (*p* < 0.001) (Table 2).

**Table 2:** Mean and SD of angular tip values for the planned (*T*1–*T*0), the achieved (*T*2–*T*0), the imprecision (|*T*2–*T*1|) and the accuracy (%) considering the individual dental groups, the individual jaws (maxilla and mandible) and the total

Arch	Tooth Group	N. observations	Tip									
			Planned (*T*1–*T*0)		Achieved (*T*2–*T*0)		Planned versus achieved	Imprecision l*T*2-*T*1l		Accuracy		Versus 100%
			Mean (°)	SD (°)	Mean (°)	SD (°)	*p* value	Mean (°)	SD (°)	Mean (%)	SD (%)	*p*-value
Maxilla	Incisor	77	3.81	15.76	3.05	13.24	< 0.001*	1.96	2.13	80.72	6.34	< 0.001*
	Canine	45	0.36	11.98	0.17	9.74	< 0.001*	1.97	1.35	78.63	6.62	< 0.001*
	Premolar	71	− 0.31	10.21	− 0.28	7.91	< 0.001*	1.93	1.39	77.52	5.18	< 0.001*
	Molar	82	9.26	10.92	7.35	8.51	< 0.001*	2.65	1.86	78.47	6.91	< 0.001*
Mandible	Incisor	81	1.64	6.87	1.29	5.28	< 0.001*	1.37	0.96	78.16	6.06	< 0.001*
	Canine	38	− 0.65	9.41	− 0.59	7.33	< 0.001*	1.86	1.09	77.42	7.29	< 0.001*
	Premolar	77	− 3.35	7.76	− 2.55	6.13	< 0.001*	1.63	0.98	77.75	5.59	< 0.001*
	Molar	74	− 6.47	8.51	− 5.03	6.65	< 0.001*	1.99	1.46	78.11	5.42	< 0.001*
Maxilla		275	3.81	13.01	3.01	10.53	< 0.001*	2.16	1.78	78.88	6.37	< 0.001*
Mandible		270	− 2.32	8.54	− 1.81	6.67	< 0.001*	1.68	1.16	77.93	5.93	< 0.001*
Total		545	0.76	11.43	0.62	9.14	< 0.001*	1.92	1.52	78.41	6.17	< 0.001*

*The mean value of accuracy was compared with a hypothetical 100% (*p* < 0.05 considered as significant)

### Rotation

Rotation too was affected by a significant difference between planned and achieved movements (*p* < 0.001) in all cases. The average total accuracy was 77.99% ± 6.58%, while that of each individual tooth group ranged from a maximum of 80.72% ± 6.34% (maxillary incisors) to a minimum of 76.59% ± 6.88% (mandibular molars). The accuracy of rotation movements was always statistically significant different (*p* < 0.001) from a hypothetical 100% (Table 3).

**Table 3:** Mean and SD of angular rotation values for the planned (*T*1–*T*0), the achieved (*T*2–*T*0), the imprecision (*T*2–*T*1l) and the accuracy (%) considering the individual dental groups, the individual jaws (maxilla and mandible) and the total

Arch	Tooth group	N. observations	Rotation									
			Planned (*T*1–*T*0)		Achieved (*T*2–*T*0)		Planned versus achieved	Imprecision l*T*2–*T*1l		Accuracy		Versus 100%
			Mean (°)	SD (°)	Mean (°)	SD (°)	p value	Mean (°)	SD (°)	Mean (%)	SD (%)	*p*–value
Maxilla	Incisor	85	− 5.58	12.99	− 4.61	10.46	< 0.001*	2.19	1.99	80.13	8.19	< 0.001*
	Canine	46	− 4.07	17.62	− 3.04	14.14	< 0.001*	2.93	2.39	78.21	6.89	< 0.001*
	Premolar	83	0.46	15.84	0.43	12.33	< 0.001*	2.81	2.37	77.49	6.08	< 0.001*
	Molar	84	4.28	17.48	3.57	14.22	< 0.001*	2.55	1.83	78.05	5.67	< 0.001*
Mandible	Incisor	81	− 5.84	10.54	− 4.65	8.46	< 0.001*	2.12	1.36	78.64	5.76	< 0.001*
	Canine	43	− 16.01	13.21	− 12.64	10.65	< 0.001*	3.78	2.45	78.47	6.07	< 0.001*
	Premolar	85	9.36	12.62	7.42	9.93	< 0.001*	3.01	1.79	76.64	6.15	< 0.001*
	Molar	81	0.39	9.07	0.31	7.24	< 0.001*	1.82	0.99	76.59	6.88	< 0.001*
Maxilla		298	− 0.88	16.29	− 0.65	13.05	< 0.001*	2.58	2.28	78.51	6.82	< 0.001*
Mandible		290	− 1.15	14.05	− 0.91	11.17	< 0.001*	2.54	1.75	77.46	6.29	< 0.001*
Total		588	− 1.01	15.21	− 0.78	12.15	< 0.001*	2.56	2.04	77.99	6.58	< 0.001*

*The mean value of accuracy was compared with a hypothetical 100% (*p* < 0.05 considered as significant)

### Tooth group comparison

A comparison of the accuracy among the different tooth groups via the Levene test was found to be statistically significant for both torque (*p* < 0.001) and tip (*p* = 0.04) movement, which is why we proceeded to the robust Brown–Forsythe version of ANOVA. Given the rejection of the null hypothesis of equality among the means with the latter test (*p* =  < 0.001 for torque and *p* = 0.05 for tip), pairwise comparisons were subsequently conducted using Tamhane's post-hoc.

This yielded statistically significant differences in torque accuracy for all but the following eight pairwise comparisons: maxillary incisor vs. maxillary canine (*p* = 0.756), mandibular incisor (*p* = 0.223) vs. mandibular canine (*p* = 0.756); maxillary canine vs. mandibular incisor (*p* = 0.892) and mandibular canine (0.990); maxillary premolar vs. mandibular premolar (*p* = 1); maxillary molar vs. mandibular molar (*p* = 1); and mandibular incisor versus mandibular canine (*p* = 1) (Table 4). Tip accuracy was only statistically significantly different between the maxillary incisor and maxillary premolar (*p* = 0.026) (Table 4).

**Table 4:** Statistical comparison of accuracy [|*T*2/*T*1|] × 100 between tooth type for both maxilla and mandible for each movement investigated, using Tamhane’s post hoc for torque and tip, and Fishers LDS post hoc (*p* < 0.05*)

Tooth type/arch		Torque		Tip		Rotation	
		*p*-value	Significance	*p*-value	Significance	*p*-value	Significance
Incisor-Maxilla	Canine-Maxilla	0.756	NS	0.933	NS	0.108	NS
	Premolar-Maxilla	0.000	*	0.026	*	0.009	*
	Molar-Maxilla	0.000	*	0.622	NS	0.039	*
	Incisor-Mandible	0.223	NS	0.258	NS	0.141	NS
	Canine-Mandible	0.756	NS	0.441	NS	0.174	NS
	Premolar-Mandible	0.000	*	0.067	NS	0.001	*
	Molar-Mandible	0.000	*	0.184	NS	0.001	*
Canine-Maxilla	Premolar-Maxilla	0.000	*	1.000	NS	0.554	NS
	Molar-Maxilla	0.000	*	1.000	NS	0.900	NS
	Incisor-Mandible	0.892	NS	1.000	NS	0.720	NS
	Canine-Mandible	0.990	NS	1.000	NS	0.850	NS
	Premolar-Mandible	0.000	*	1.000	NS	0.189	NS
	Molar-Mandible	0.000	*	1.000	NS	0.182	NS
Premolar-Maxilla	Molar-Maxilla	0.000	*	1.000	NS	0.580	NS
	Incisor-Mandible	0.000	*	1.000	NS	0.262	NS
	Canine-Mandible	0.000	*	1.000	NS	0.428	NS
	Premolar-Mandible	1	NS	1.000	NS	0.394	NS
	Molar-Mandible	0.000	*	1.000	NS	0.378	NS
Molar -Maxilla	Incisor-Mandible	0.000	*	1.000	NS	0.566	NS
	Canine-Mandible	0.000	*	1.000	NS	0.736	NS
	Premolar-Mandible	0.000	*	1.000	NS	0.158	NS
	Molar-Mandible	1	NS	1.000	NS	0.152	NS
Incisor-Mandible	Canine-Mandible	1	NS	1.000	NS	0.890	NS
	Premolar-Mandible	0.000	*	1.000	NS	0.049	*
	Molar-Mandible	0.000	*	1.000	NS	0.047	*
Canine- Mandible	Premolar-Mandible	0.000	*	1.000	NS	0.134	NS
	Molar-Mandible	0.000	*	1.000	NS	0.129	NS
Premolar-Mandible	Molar-Mandible	0.000	*	1.000	NS	0.968	NS

NS, not significant

As for the rotation movement, the Levene test yielded a not statistically significant result (*p* = 0.573), so the classical ANOVA was conducted, which rejected the null hypothesis of equality between the means (*p* = 0.013). In this case, subsequent pairwise comparisons were conducted using Fisher's LSD post-hoc test. This indicated statistically significant differences between six pairs, namely: maxillary incisor vs. maxillary premolar (*p* = 0.009), maxillary molar (*p* = 0.039), mandibular premolar (*p* = 0.001)] and mandibular molar (*p* = 0.001); and mandibular incisor vs. mandibular premolar (*p* = 0.049) and mandibular molar (*p* = 0.047) (Table 4).

### Linear measurements

As regards the linear intra-arch values investigated, there was high accuracy in the anterior sectors (83.54% ± 5.19% and 79.99% ± 4.26% for maxillary canines and first premolars, respectively; and 81.90% ± 3.30% and 80.05% ± 2.96% for mandibular canines and first premolars, respectively). However, accuracy significantly decreased towards the posterior sectors (73.14% ± 3.57% and 67.28% ± 4.37% for the maxillary first and second molars, respectively; and 73.43% ± 3.74% and 68.32% ± 5.99% for the mandibular first and second molars, respectively). The Friedman test yielded a statistically significant result for all investigated measures (*p* < 0.05).

Subsequent pairwise comparisons between the initial value at *T*0, the planned value (*T*1) and the one achieved (*T*2) always showed a statistically significant increase in intra-arch linear distances with respect to baseline (*T*0–*T*2), with the exception of the upper (*p* = 0.102 for IM1 and *p* = 0.359 for IM2) and lower molars (*p* = 0.359 for IM1 and *p* = 0.609 for IM2) (Table 5).

**Table 5:** Mean and SD of linear measurements for the planned (*T*1–*T*0), the achieved (*T*2–*T*0), the imprecision (l*T*2–*T*1l) and the accuracy (%)

Arch	Linear measurements	N.of observations	Linear measurements									
			Planned (*T*1–*T*0)		Obtained (*T*2–*T*0)		Imprecision |(*T*2–*T*1|)		Accuracy		*T*0 versus *T*1	*T*0 versus *T*2
			Mean (mm)	SD (mm)	Mean (mm)	SD (mm)	Mean (mm)	SD (mm)	Mean (%)	SD (%)		
Maxilla	Ic	25	2.13	1.73	1.81	1.52	0.36	0.19	83.54	5.19	< 0.001*	0.003*
	Ip1	25	3.53	2.39	2.82	1.92	0.76	0.43	79.99	4.26	< 0.001*	0.003*
	Ip2	25	3.25	2.43	2.47	1.82	0.85	0.54	76.76	4.68	< 0.001*	0.022*
	Im1	25	2.16	2.21	1.59	1.63	0.74	0.33	73.14	3.57	< 0.001*	0.102
	Im2	25	1.30	2.16	0.90	1.47	0.68	0.43	67.28	4.37	0.006*	0.359
Mandibular	Ic	25	1.86	1.68	1.52	1.38	0.39	0.23	81.90	3.30	< 0.001*	0.009*
	Ip1	25	2.72	1.72	2.19	1.39	0.56	0.30	80.05	2.96	< 0.001*	0.003*
	Ip2	25	2.47	2.41	1.92	1.89	0.61	0.46	76.80	3.65	< 0.001*	0.022*
	Im1	25	1.15	1.98	0.84	1.46	0.50	0.34	73.43	3.74	0.006*	0.359
	Im2	25	0.79	1.76	0.53	1.17	0.53	0.39	68.32	5.99	0.033*	0.609

Pairwise comparisons between *T*0, *T*1 and *T*2 are reported according to Friedman’s test (*p* < 0.05 considered as significant)*

## Discussion

A good orthodontic treatment performed with lingual appliances begins with accurate set-up, which is particularly important in lingual orthodontics due to the great heterogeneity of the lingual surface of the teeth [19]. Thankfully, recent technological innovations allow effective digital set-up via a method that is more streamlined and facilitated than manual set-up. Furthermore, planned overcorrections are easily quantifiable, making the individualization of the entire orthodontic treatment very precise [22].

The introduction of the passive lingual self-ligating bracket with square-slot in 2011 made the clinician's experience in performing archwire ligating less decisive. The square slot keeps the archwire within it even during derotation movements and retraction of anterior teeth. When using a full-thickness lingual archwire, the same minimal wire–slot play applies in both second and third order, making dimensional control of each tooth more efficient.

The study presented here aimed to investigate the combined effectiveness of the digital set-up and the new passive lingual self-ligating bracket with square slot, quantifying the clinical accuracy of achieving the result planned in the digital set-up as a percentage. This analysis would lay the foundations for identifying any overcorrections to be included in the set-up both as regards angular values (torque, tip and rotation) and transverse linear intra-arch measurements.

We calculated the accuracy for the various movements by tooth group in each arch since anatomical differences at the root level influence the resistance to orthodontic movement [29]. Resistance is also influenced by the position of the tooth in the arch and the arch itself. Specifically, the lower arch usually has a more compact bone, which offers greater resistance to dental movement [30].

The results of this study highlight a common trend, namely a decreasing accuracy in angular measurements (torque) and transverse linear intra-arch measurements from the front to the back of the arch. While torque movements were > 81% accurate in the anterior sectors (incisors and canines), they were significantly reduced, at < 70%, in the molar areas; similarly, the accuracy of linear intra-arch measurements was > 81% in the anterior sectors and < 69% in the posterior ones. The same trend is perceptible when analysing both tip measurements in the maxillary arch, albeit to a far lesser extent, with < 2% differences in accuracy between the anterior and posterior sectors, and rotations in both arches (< 2%). As for the accuracy of the tip in the mandibular arch, the accuracy values for the anterior and posterior sector were comparable.

The differences in the accuracy of torque and linear intra-arch measurements can be explained by the different root morphology of the various tooth groups analysed (single-rooted teeth are easier to move than the multi-rooted teeth) [29] and by the different bone anatomy of the various arch sectors [30]. In addition, the posterior sector bracket slots are slightly oversized compared to the nominal size. This is to avoid excessive friction and facilitate sliding mechanics, particularly useful in extraction cases [20], but it does negatively affect torque expression.

Another factor to consider is that in the terminal portions of the arch the archwire is more flexible, exerting the so-called "trampoline effect", which limits the transmission of orthodontic forces [23], not to mention the influence that masticatory forces could have at this level.

It should also be noted that the appliance investigated is characterized by vertical insertion of the archwire via a sliding-door mechanism in the front sectors and a hinge-cup mechanism in the rear sectors. This, in turn, could affect the accuracy of the torque, as twisting the archwire inside the slot could force the hinge-cup system in the posteriors sectors, causing a loss of information. That being said, the accuracy of the tip, which in this study instead remained constant in both arches progressing from the front to the back sector, would seem not to support this hypothesis. The conclusion therefore is that torque in itself is a more difficult movement to achieve than tip and rotation. In fact, torque movements displayed high accuracy, despite the very limited design of the bracket, both in the upper arch (1.5 mm mesiodistal direction) and in the lower arch (1.2 mm) [17] as did rotation. In the latter case, the use of a full-thickness wire would seem to be fundamental.

These findings are in line with those of Albertini et al., although they investigated the use of a conventional lingual bracket with rectangular slot (0.018 × 0.025-in.) and found a slightly greater accuracy for angular movements [23]. These differences could be explained by the imprecision inherent in the measurement method used in both studies. Our results are also similar to those of Grauer and Proffit [24] and Pauls 2010 [25, 26], who found rotation discrepancies of less than 4° and 5°, respectively.

As far as linear measurements are concerned, our study yielded differing results from those reported by both Albertini et al. [23] and Grauer and Profitt [24]. Specifically, we found less expansion at the second molars (about 2/3 of that planned), while both Albertini et al. [23] and Grauer and Profitt [24] showed a contraction at this level. However, as pointed out by previous authors, these differences could be due to the preferential use of elastic power chains over that of continuous metal ligatures. This would lead to a constriction of the arch and to the horizontal bowing effect, not effectively counteracted by the rigidity of the lingual arch-wire, which is smaller than that used in vestibular orthodontics [20].

Although this is the first study conducted on this method, it does have a major limitation, namely its retrospective design. Future randomized clinical trials with a control group treated by the same operator using conventional lingual appliances are warranted in order to obtain conclusive findings. In addition, this study involved the treatment of non-extraction cases of moderate complexity; future researches with the inclusion of extraction cases and addition of overcorrections in the digital set-up would provide more informations.

## Conclusions

The study showed that:

- The combined use of the digital set-up and self-ligating lingual brackets with square slot demonstrates relative high accuracy in terms of both angular and linear measurements.
- Both torque and linear movements were highly accurate in the anterior sectors, but this decreased in the posterior sectors.
- Tip and rotation movements displayed high accuracy in both the anterior and posterior sectors.
- Overcorrection should be included in the set-up to fill the inaccuracy gap evidenced, especially as regards torque and expansion of the posterior sectors.

## Data Availability

Not applicable.

## Abbreviations

LSP: Lingual straight plane
Cu-NiTi: Copper nickel titanium
TMA: Titanium molybdenum alloy
